# Extracellular Na^+^ levels regulate formation and activity of the Na_X_/alpha1-Na^+^/K^+^-ATPase complex in neuronal cells

**DOI:** 10.3389/fncel.2014.00413

**Published:** 2014-12-04

**Authors:** Emmanuelle Berret, Pascal Y. Smith, Mélaine Henry, Denis Soulet, Sébastien S. Hébert, Katalin Toth, Didier Mouginot, Guy Drolet

**Affiliations:** ^1^Centre de Recherche du CHU de Québec, Axe NeurosciencesQC, Canada; ^2^Faculté de Médecine, Département de Psychiatrie et Neurosciences, Université LavalQC, Canada; ^3^Institut Universitaire de Santé Mentale de Québec, Université LavalQC, Canada

**Keywords:** sodium, Na_X_ channel, Na^+^/K^+^-ATPase, median preoptic nucleus, salt diet

## Abstract

MnPO neurons play a critical role in hydromineral homeostasis regulation by acting as sensors of extracellular sodium concentration ([Na^+^]_out_). The mechanism underlying Na^+^-sensing involves Na^+^-flow through the Na_X_ channel, directly regulated by the Na^+^/K^+^-ATPase α1-isoform which controls Na^+^-influx by modulating channel permeability. Together, these two partners form a complex involved in the regulation of intracellular sodium ([Na^+^]_in_). Here we aim to determine whether environmental changes in Na^+^ could actively modulate the Na_X_/Na^+^/K^+^-ATPase complex activity. We investigated the complex activity using patch-clamp recordings from rat MnPO neurons and Neuro2a cells. When the rats were fed with a high-salt-diet, or the [Na^+^] in the culture medium was increased, the activity of the complex was up-regulated. In contrast, drop in environmental [Na^+^] decreased the activity of the complex. Interestingly under hypernatremic condition, the colocalization rate and protein level of both partners were up-regulated. Under hyponatremic condition, only Na_X_ protein expression was increased and the level of Na_X_/Na^+^/K^+^-ATPase remained unaltered. This unbalance between Na_X_ and Na^+^/K^+^-ATPase pump proportion would induce a bigger portion of Na^+^/K^+^-ATPase-control-free Na_X_ channel. Thus, we suggest that hypernatremic environment increases Na_X_/Na^+^/K^+^-ATPase α1-isoform activity by increasing the number of both partners and their colocalization rate, whereas hyponatremic environment down-regulates complex activity via a decrease in the relative number of Na_X_ channels controlled by the pump.

## Introduction

Sodium (Na^+^) and more specifically sodium chloride (NaCl) is the most abundant extracellular electrolyte and the major determinant of osmolarity. Because sodium homeostasis is essential for life, Na^+^ concentration ([Na^+^]) in the plasma and cerebrospinal fluid (CSF) needs to be permanently monitored in order to maintain a physiological set point (Verbalis, 2003). Previously, it has been shown that specific Na^+^-sensors exist in the brain, particularly in the circumventricular organs (CVOs) and in the organs surrounding the third ventricle (Cox et al., 1987; Denton et al., 1996; McKinley et al., 2003). The median preoptic nucleus (MnPO), a main Na^+^-sensor region, is located in the lamina terminalis along the third ventricle and holds a strategic location giving MnPO neurons direct access to ionic composition of the CSF, and a key position to detect and regulate changes in [Na^+^] (Fitzsimons, 1998; Hussy et al., 2000; Grob et al., 2004). It has been proposed that extracellular sodium ([Na^+^]_out_) variations are measured mainly by an atypical sodium channel, namely Na_X_, a recognized Na^+^-sensor (Hiyama et al., 2002, 2004; Grob et al., 2004; Noda, 2006). Moreover, 80% of MnPO neurons express this atypical sodium channel (Voisin et al., 2012). Specifically, Na_X_ channel is a Na^+^ leak channel allowing Na^+^ ions to pass through the membrane, and detect local variations in [Na^+^]_out_ (Tremblay et al., 2011). Previous study described a direct interaction between the Na_X_ channel and the Na^+^/K^+^-ATPase pump in astrocytes located in the CVOs in mice (Shimizu et al., 2007). However, Nehme et al., have showed that the expression pattern of Na_X_ channels in mice and rat is different, this could lead to different mechanism of action in those species (Nehmé et al., 2012). Recently, we demonstrated that Na_X_ channel is directly regulated by α-1 isoform of the Na^+^/K^+^-ATPase, forming a functional complex located close to the cell membrane and plays an important role in the regulation of local [Na^+^] (Berret et al., 2013). However, the mechanism by which other factors modulate the activity of this functional complex remains unknown.

It is well established that environmental factors affect or regulate functional systems in an organism. For instance, low-salt intake can induce hyponatremia and hypovolemia as well as a hyper-activity of specific neuronal population sensitive to aldosterone in Sprague Dawley rats (Geerling et al., 2006; Geerling and Loewy, 2008). In the other hand, high-salt intake can increase sympathetic hyperactivity and arterial pressure in animal models of hypertension such as Dahl Salt-sensitive rats and spontaneous hypertensive rats but not in their respective normotensive controls, Dahl Salt-resistant, WKY rats (Huang and Leenen, 1995, 1998, 2002), as well as in Wistar rats (Rahmouni et al., 2002; Mouginot et al., 2007). These differences suggest a mechanism allowing the maintenance of Na^+^ homeostasis and arterial pressure in rats that are not sensitive to high-salt intake. To date, however, how environmental changes in [Na^+^] influence Na^+^-sensing in the brain remains poorly understood.

In this study, we tested the hypothesis that activity of the Na_X_/Na^+^/K^+^-ATPase α-1 complex is regulated by systemic changes in extracellular [Na^+^]. Wistar rats were fed with high, normal, or low-salt diets. Electrophysiological recordings were performed on dissociated MnPO neurons, providing an *in vivo* readout of Na^+^-sensing in the brain. These studies demonstrated that the activity of the complex could be upregulated and downregulated in hypernatremic and hyponatremic conditions, respectively. We also developed a neuronal cell culture system (mouse Neuro2a cells) to dissect the molecular mechanisms supporting these observations.

## Materials and methods

The experiments performed in the present study were in accordance with the guidelines established by the Canadian Council on Animal Care, and were duly approved by our institutional Animal Care Committee (CPAC/Université Laval). Young male Wistar rats were obtained from Charles River Canada (St-Constant, Québec) and housed in plastic cages (two rats per cage) for 1 week of acclimatization to standard laboratory conditions before use for experimentation (cycle of 14 h of light and 10 h of dark at 23°C).

### Experiments 1: dissociated MnPO neurons

#### Experimental model

***Animal diets***. Wistar rats (3 weeks old, male, from Charles River) were randomly assigned to receive either regular Na^+^ diet (0.6% NaCl-10 animals; 2 weeks), high salt-diet (8% NaCl-10 animals; 1 week) or low-salt diet (0.02% NaCl-10 animals). In order to avoid neophobia, all rats receiving high or low-salt diet were fed with this diet concomitantly with regular diet, 48 h before starting the high or low-salt diet alone. All the animals had access to tap water ad libidum during the diet. Na^+^ diets were based on previously published studies (Geerling et al., 2006; Mouginot et al., 2007) to allow a change in basal CSF [Na^+^].

***Isolation of primary MnPO neurons***. Wistar rats were deeply anesthetized by intraperitoneal injection of a ketamine-xylasine mixture (87.5 and 12.5 ml/kg, respectively) and decapitated. The brain was removed from the skull and immersed in oxygenated (95% O_2_-5%CO_2_) ice-cold (4°C) dissection solution containing (in mM): 200 sucrose, 10 D-Glucose, 2 KCl, 1 CaCl_2_, 3 MgCl_2_, 26 NaHCO_3_, and 1.25 NaH_2_PO_4_, and a sagittal slice of 350 μm containing the MnPO was prepared. The ventral region of the MnPO was then punched out and placed in oxygenated artificial cerebrospinal fluid (aCSF) containing (in mM): 140 NaCl, 3.1 KCl, 2.4 CaCl_2_, 1.3 MgCl_2_, 10 HEPES, 10 D-Glucose, pH 7.4 (adjusted with NaOH), osmolality: 295–300 mosm.kg^−1^. Enzymatic dissociation of the MnPO micropunchs was obtained by successively incubating the pieces of tissue in aCSF containing pronase (0.1 mg/mL) for 10 min at 37°C, then in aCSF containing Bovin Serum Albumin (BSA, 2 mg/mL) for 15 min at 37°C and finally in aCSF containing thermolysin (0.1 mg/mL) for 10 min at 37°C. The pieces of tissue were then mechanically dissociated by trituration using glass Pasteur pipettes with reduced diameter. Centrifugation (3500 rpm/3 min at room temperature) was performed and neurons were re-suspended in aCSF solution. 50 μL of aCSF containing dissociated cells was directly platted on laminin-coated micro cover glasses. The seeded micro cover plate were incubated during 1 h at 37°C in a 100% O_2_ humidified atmosphere, before being washed for keeping cells attached only. Finally neurons were used for patch-clamp recordings, or immunocytochemistry, or trypan blue test.

#### Electrophysiological recordings

***Standard conditions***. Whole-cell voltage clamp recordings were performed on cultured or dissociated neurons visualized under the Hoffman modulation contrast. Micropipettes were filled with a solution containing (in mM): 130 K-Gluconate, 10 HEPES, 6 NaCl, 0.3 Na^+^-GTP, 4 Na^+^-ATP, 10 EGTA, pH 7. 2, osmolality: 295–300 mosm.kg^−1^. Micropipettes had a resistance of 4–5 MΩ. The extracellular solution (control aCSF) had the following composition (in mM): 140 NaCl, 3.1 KCl, 2.4 CaCl_2_, 1.3 MgCl_2_, 10 HEPES, 10 D-Glucose, pH 7.4, osmolality: 295–300 mosm.kg-1.Cells were voltage clamped at −60 mV with an EPC8 amplifier (HEKA). Recorded cells were randomly chosen. Hypernatremic aCSF was obtained by increasing NaCl concentration to 170 mM. Note that Na^+^ sensors neurons are defined as ones responding to hypernatremic application with a minimal inward current of 4 pA. To measure the Na^+^ current we used the Labchart software to determine the window box taking in count noise; the size of the window was identical in RMP and Peak measurements. The condition allows separating the Na^+^ response of the membrane background noise. To test the effect of sodium environment or sodium diet on Na_X_/Na^+^/K^+^-ATPase complex, pharmacological test were performed, and stock solution of strophanthidin (50 mM) (a specific inhibitor of α-1 isoform of the Na^+^/K^+^-ATPase pump) were directly diluted in control aCSF.

***Channel conductance and permeability measurements***. For one set of experiments only designed to test the Na^+^ conductance and Na^+^ permeability of the Na_X_ channel (**Figures 2B,C**), the micropipettes were filled with a solution containing (in mM): 100 NaCl, 25 CsCl, 5 HEPES, 5 TEACl, 0.3 Na^+^-GTP, 4 Na^+^-ATP, pH 7.2, osmolality: 295–300 mosm.kg^−1^ (adjusted with mannitol). Isonatremic aCSF required to test Na^+^ permeability and Na^+^ conductance of the channel had the following composition (in mM): 100 NaCl, 3.5 CsCl, 3.1 KCl, 2.4 CaCl_2_, 1.3MgCl_2_, 0.3 CdCl, 10 HEPES, 10 TEACl, 5 D-Glucose, 1 4-aminopyridine, and 0.0005 TTX, pH 7.4, osmolality: 295–300 mosm.kg^−1^ (adjusted with mannitol). To obtain the reversal potential of the [Na^+^]-induced currents (ENa^+^), all the current traces were subtracted from the total leak current by scaling the zero-current potential of the ramp-activated current to 0 mV under the isonatriruric condition (control). Recorded cells were randomly chosen. Hypernatremic aCSF was obtained by raising NaCl concentration to 170 mM. All the electrophysiological recordings performed in dissociated neurons and Neuro2a cells were carried out at room temperature (21–23°C).

***Calculation of the Na^+^ conductance and the Na^+^ permeability***. Calculation of the Na^+^ conductance (g_Na_) was obtained from modified Hodgkin and Huxley equation as described in Berret et al. (2013). Calculation of the permeability (P_Na_) was obtained from modified GHK equation as described in Berret et al. (2013).

### Experiments 2: neuro2a cells culture

#### Experimental model

***Cell line***. Mouse Neuroblastoma 2a (Neuro2a) cells were cultured in Dulbecco's modified Eagle's medium (DMEM) supplemented with 10% fetal bovine serum. Neuro2a cell differentiation was induced by incubating cells with DMEM free-serum during 48 h.

***Cell Transfection***. 250,000 Neuro2a cells grown plated on round microscope coverslip in 6-well plates. The next day, cells were transfected with 100 nM of small interfering RNAs (siRNAs) against Na_X_ or α1 isoform of the Na^+^/K^+^-ATPase (ON-TARGETplus SMART pool siRNA Scn7a or ATP1a1, Dharmacon) or scrambled sequence sense or antisense (Dharmacon, Lafayette, CO, USA) using LipofectAMINE 2000 following the manufacturer's instructions.

***Cell Na^+^ environment***. For experiments requiring Na^+^ concentration changes, Neuro2a cells were plated on round microscope coverslip in 6-well plates with different [Na^+^] in DMEM during 48 h. DMEM with 145 mM NaCl (normal DMEM) has been used as control environment, and DMEM with a lower (135 mM NaCl) or a higher (155 mM NaCl) [Na^+^] has been used as hypo- or hypernatremic environment respectively. These values represent physiological variations in [Na^+^] (Huang et al., 2004).

#### RNA extraction and quantitative RT-PCR (qRT-PCR)

RNA was extracted from cells using Trizol Reagent (Invitrogen, Lifes Technologies) according to the manufacturer's instructions. 1 μg of RNA was reverse transcribed using the iScript cDNA Synthesis Kit (Bio-Rad). The complementary DNA was amplified using qPCR SsoFast Evagreen Supermix (Bio-Rad) and analyzed in the LightCycler 480 II (Roche Applied Science). For Na_X_ mRNA analysis, TaqMan Gene Expression assay (ID # Mm008801952_m1) was used and normalized with the endogenous control GAPDH (ID# Mm99999915_g1).

Oligonucleotides used for ATP1a1 gene (Na^+^/K^+^-ATPase α1 isoform) were CTTTCTTATCCTACTGCCCCG forward and ATAATGAGCTTCCGCACCTC reverse. RPL32 was used as endogenous telltale to quantify ATP1a1 level. Oligonucleotides for RPL32 were TTAAGCGAAACTGGCGGAAAC forward and TTGTTGCTCCCATAACCGATG reverse.

#### Immunocytochemistry

The round microscope coverslips with Neuro2a were removed from 6-well plates to be fixe for 60 min in a paraformaldehyde (PFA) solution and immediately used for immunocytochemistry. The fixed cells were incubated overnight at 4°C in PBS 1X containing 5% native goat serum, 1% BSA with rabbit anti-Na_X_ antibody targeting the interdomain 2–3 region of the Na_X_ channel's α-subunit (1/250) and mouse anti-NeuN (1/500, Clone A60, Millipore) or mouse anti-Na^+^/K^+^-ATPase α3 subunit (1/10, Clone XVIF9-G10, Sigma-Aldrich) or mouse anti-Na^+^/K^+^-ATPase α1 subunit (1/10, Clone C464.6, Millipore) or chicken anti-MAP-2 antibody (1/100, Polyclonal Antibody, Millipore). The cells were first washed in PBS and incubated for 2 h at room temperature in PBS 1Xcontaining AlexaFluor-488 goat anti-rabbit (1/500, green, Invitrogen), and AlexaFluor-555 goat anti-mouse (1/500, red, Invitrogen) or AlexaFluor-555 goat anti-chicken (1/500, red, Invitrogen) as secondary fluorescent antibodies to visualize the Na_X_ and NeuN or Na^+^/K^+^-ATPase α3 or α1 isoforms or MAP-2 proteins, respectively. The specificity of the Na_X_ antibody has been tested using SCN7A control peptide (Cedarlane; 68912-12) at different concentration from 0 to 10 μg in differentiated Neuro2a cells (see Supplemental Figure 1).

#### Confocal microscopy and image analysis

Confocal laser scanning microscopy was performed with an IX81-ZDC microscope equipped with a FV1000 scanning head and an Olympus 60X OSC NA 1.4 objective lens. Confocal images were acquired by sequential scanning with the 405, 488, and 546 nm laser lines and the variable bandwidth filters were set optimally according to the spectral properties for DAPI, AlexaFluor 488 and AlexaFluor 555 dyes. The Fluoview imaging software ASW3.01a (Olympus America Inc, Melville, NY) was used to acquire and export the z-stacks. Maximum intensity projections and volume rendering were calculated using the Surpass module in Bitplane Imaris 7.5.1 (Zurich, Switzerland). Colocalization analysis was performed with Bitplane Imaris 7.5.1 colocalization module using the Costes' estimation for automatic threshold, which compares the Pearson's coefficient for non-randomized vs randomized images and calculates the significance (Costes et al., 2004). Colocalization channel of Na_X_ with Na^+^/K^+^-ATPase α1 isoform was generated for visual representation, and Pearson's coefficients were calculated. Sum of the intensities of Na_X_ with Na^+^/K^+^-ATPase α1 were calculated per cell volume using Bitplane Imaris Cell 7.5.1 module.

Here we provide a detailed explanation of the Coste's estimation we used to determine the intensities of the Na_X_ and Na^+^/K^+^-ATPase α1 respective signals: The Costes's estimation is used to estimate the background, i.e., to calculate the probability that channel thresholding levels are not set too low during the colocalization process based on the theoretical point spread function and the voxel size of the image stack. Random images are generated by smoothing white noise with a gaussian point spread function of a specified width based on the voxel size. The software calculates the regression line of 2D scatter plots for each Z plane. Then, the position is decreased over the regression line until the Pearson coefficient of the background is zero. This allows to gate automatically the signal for each channel, without the intervention of the operator (no bias). The random noise is removed from the colocalization analysis. This method allows the generation of a significant colocalization channel. Then, this colocalization channel is used to generate a 3D model (3D mesh using marching cube algorithm in Imaris Surpass module). Thereafter, the calculation of the sums of the intensities of the channels of interest is calculated within the volume of the 3D mesh using the Measurement Pro module in Imaris (integration of the signal within the volume). This is a straightforward approach to segment the volume and to calculate the quantity/ratio of fluorescent materials that are truly colocalized.

#### Electrophysiological recordings

Parameters and solutions used to perform electrophysiological recordings on Neuro2a cells and on dissociated cells from MnPO previously described were identical.

### Statistical analysis

Results obtained from electrophysiological recordings and molecular analysis were expressed as means ± SE. Current amplitude was normally distributed and analyzed using parametric statistical tests. Comparisons of strophanthidin effect in Neuro2a cells under control condition was performed using paired Student's *t*-test, while strophanthidin inhibition comparison under SCR or siRNA conditions was performed using paired Student's *t*-test. Comparison between 135, 145, 155 mM NaCl condition, and between low-, control and high-salt diets (basal response tests, strophanthidin tests and mRNA expression) were performed using One-Way analysis of variance (ANOVA) as indicated in the text. We used the Newman-Keuls comparison test as a *post-hoc* test for correcting for multiple comparisons. Statistical significance was defined as *P* < 0.05.

## Results

### Changes in systemic Na^+^ influence Na_X_/Na^+^/K^+^-ATPase α-1 complex activity in MnPO neurons

**Figure 1:**
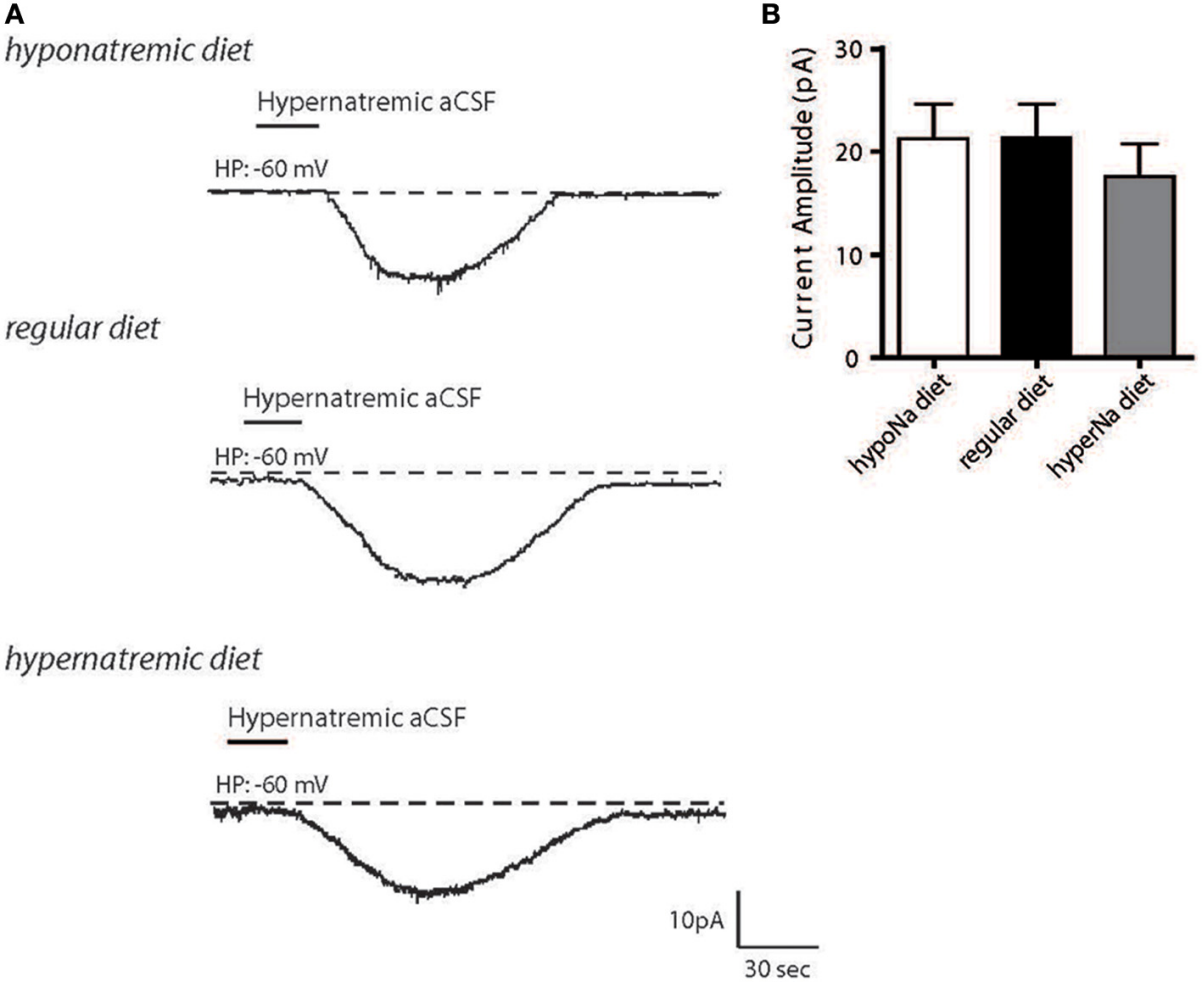
**Hypernatremic diet do not induced changes in the basal response to a transient change in [Na^+^]_out_ in dissociated MnPO neurons**. Typical representation of the inward Na^+^ current evoked by hypernatremic (170 mM NaCl) aCSF in dissociated MnPO neurons after hyponatremic diet (top trace), under standard condition (regular diet, middle trace) or hypernatremic diet (bottom trace) **(A)**. Bar graph showing different characteristics of the basal response triggered by application hypernatremic aCSF under these three conditions for Na^+^ current amplitude **(B)**.

We first aimed to determine how changes in Na^+^ intake could influence the activity of the Na_X_/Na^+^/K^+^-ATPase α-1 complex in MnPO neurons. Wistar rats were fed with high- or low-salt diets, the MnPO neurons were isolated, and we measured the neuronal basal response to the transient application of hypernatremic (170 mM NaCl) artificial CSF (aCSF) (Figure 1A). We observed no significant changes in inward Na^+^ current amplitude following high- or low-salt diets [21.25 ± 3.36, 21.29 ± 3.30, 17.60 ± 3.13 pA for low-, control and high-salt diet respectively; ANOVA *F*_(2,36)_ = 0.4458; *P* = 0.6438, *n* = 13 for each condition, Figure 1B]. This result shows that changes in salt intake does not modulate the amplitude of the basal response to external Na^+^ transient variations and that cell resistance remained unaltered. Thus, changes in systemic Na^+^ seems not alter the basal Na^+^-sensing ability.

**Figure 2:**
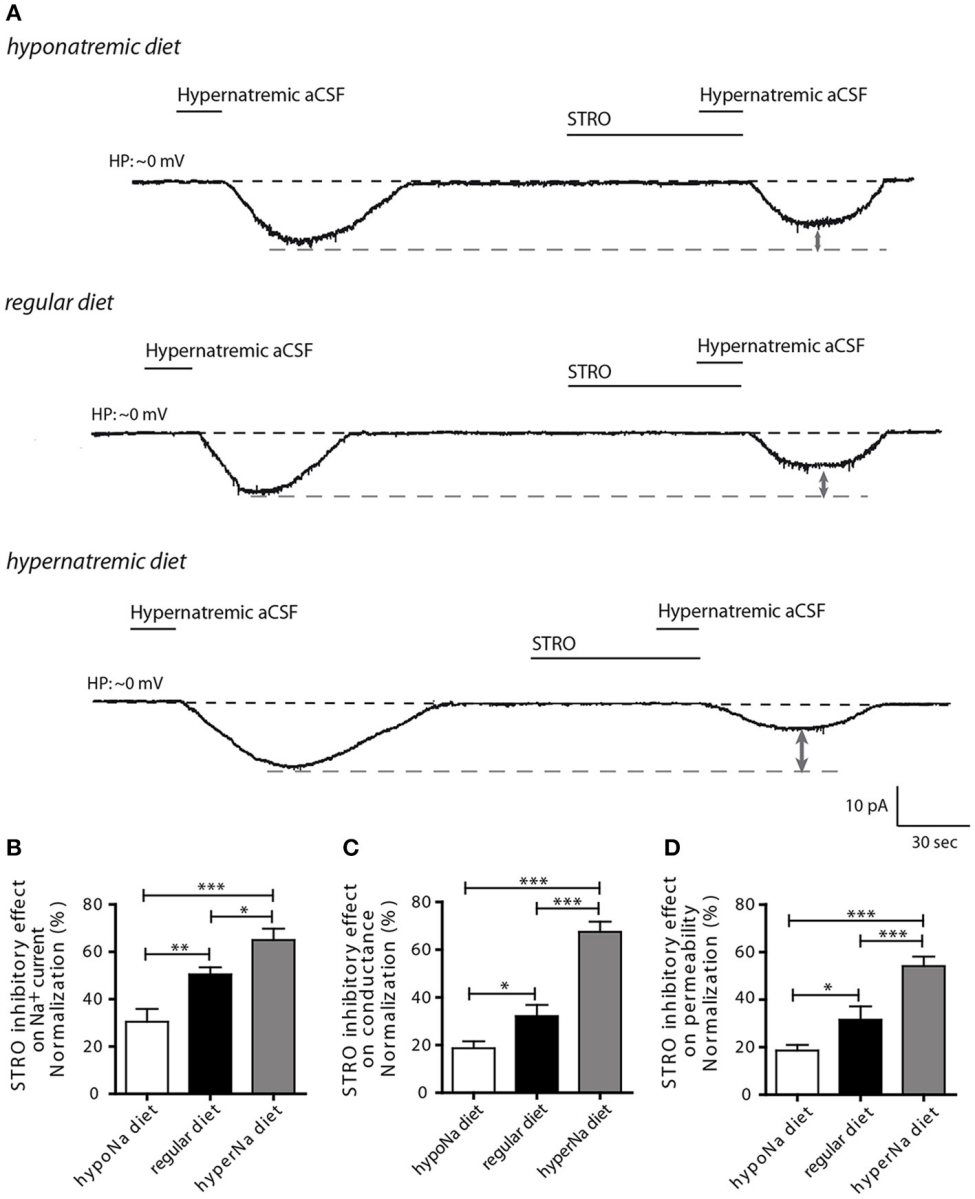
**The influence of hypo- and hypernatremic diets on strophanthidin inhibitory effect in dissociated MnPO neurons**. **(A)** Inward Na^+^ current triggered by hypernatremic(170 mM NaCl) aCSF under control condition and during application of strophanthidin (40 μM/1 min) under the three conditions (top trace: hyponatremic diet, middle trace: standard diet, bottom trace: hypernatremic diet). Normalized strophanthidin inhibitory effect on the inward Na^+^ current amplitude **(B)**, on Na^+^ current conductance **(C)** and on Na^+^ current permeability **(D)** evoked by hypernatremic aCSF in dissociated MnPO neurons under the three conditions. (^*^P < 0.5; ^**^P < 0.1; ^***^P < 0.01).

Next, we aimed to determine whether Na_X_ regulation by the Na^+^/K^+^-ATPase α-1isoform could be influenced by low or high salt diets. Previously, we demonstrated that Na^+^/K^+^-ATPase α-1 isoform regulates Na^+^ current flow through the Na_X_ channel by modulating its permeability to Na^+^ ions. This mechanism was demonstrated by changes in Na^+^ conductance and permeability measurements (Berret et al., 2013). To address this, we investigated the effect of strophanthidin-mediated inhibition on inward Na^+^ current triggered by a transient application of hypernatremic aCSF. We applied 40 μM of strophanthidin for 1 min, the effective dosage MnPO neurons was determined from the dose-response curves shown in Berret et al., 2013 (Figure 2A). We observed a significant reduction of strophanthidin-mediated inhibition after low salt intake, whereas, a significant increase was observed after high salt intake [30.43 ± 5.42, 50.41 ± 2.98, 64.93 ± 4.83% for low-, control and high-salt diet respectively; ANOVA *F*_(2,28_) = 14.69; *P* = < 0.0001, *n* = 10] (Figure 2B). These data demonstrate that low or high salt diets modulate strophanthidin-mediated inhibition.

To further characterize this process, we next applied depolarizing voltage ramps (from −10 to 20 mV_16 mV/s) to examine the effects of strophanthidin-mediated inhibition on Na^+^ conductance (g*_Na_*) and permeability (p_Na_). Strophanthidin-mediated inhibition on g*_Na_* was significantly reduced after a low-salt diet, while this inhibition was significantly increased after a high-salt diet [18.68 ± 2.92, 32.17 ± 4.66, 67.40 ± 4.27% for low-, control, high-salt diet respectively; ANOVA *F*_(2,24)_ = 39.08; *P* = < 0.0001, *n* = 9, Figure 2C]. Similar results were observed with Na^+^ permeability [18.61 ± 2.38, 31.56 ± 5.61, 54.09 ± 3.96% for low-, control and high-salt diet respectively; ANOVA *F*_(2,24)_ = 18.26; *P* = < 0.0001, *n* = 9, Figure 2D]. The modulation of these two membrane parameters is consistent with the strophanthidin-mediated inhibition on inward Na^+^ current. Together, these results demonstrate that a low-salt diet negatively impairs the control of Na^+^/K^+^-ATPase α-1 isoform on Na_X_ permeability, whereas, a high-salt diet increases the control of Na^+^/K^+^-ATPase α-1 isoform on Na_X_ permeability.

Thus, this first set of experiments suggest that changes in environmental systemic [Na^+^] must participate on the regulation of Na_X_/Na^+^-K^+^-ATPase α-1 complex activity without affecting the basal Na^+^ sensing.

### Differentiated neuro2a cells provide a novel cellular model to study Na^+^ homeostasis

**Figure 3:**
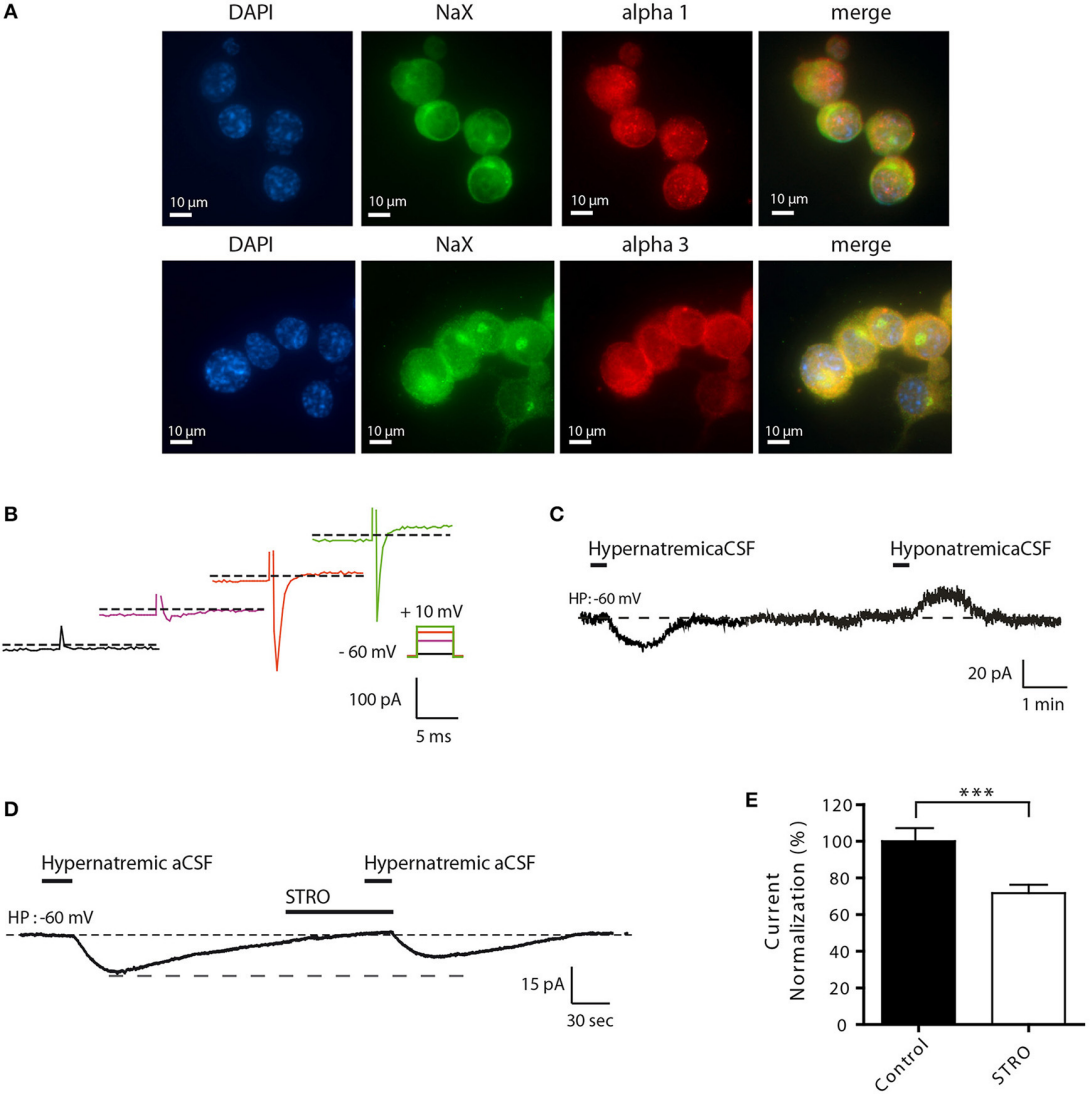
**Differentiated Neuro2a cells display all the characteristics of a neuronal Na^+^-sensor**. **(A)** Immunocytochemical staining of DAPI, Na_X_ channel, and the Na^+^/K^+^-ATPase α-1 or α-3 (upper and lower panel respectively) in differentiated Neuro2a cells. **(B)** Illustration of voltage-activated Na+ current recorded in the voltage-clamp mode in differentiated Neuro2a cells in response to depolarizing voltage steps (−55, −25, −5, 10 mV). **(C)** Inward Na^+^ current triggered by hypernatremic (170 mM NaCl) and hyponatremic (100 mM NaCl) aCSF in differentiated Neuro2a cells. **(D)** Strophanthidin-mediated inhibition of inward Na^+^ current in differentiated Neuro2a cells. **(E)** Normalized Na^+^ current amplitude triggered by the application of hypernatremic aCSF under control condition and during strophanthidin application (40 μM). (^***^P < 0.01).

To better understand the molecular mechanisms involved in this process, we developed a novel cell culture model using differentiated Neuro2a cells. This strategy was used because of various technical limitations associated with the study of isolated MnPO neurons (e.g., low cell number, viability, and transfection efficiencies). We first demonstrated that Neuro2a cells can be used as a model to study the effect of environmental Na^+^ changes on the Na_X_/Na^+^/K^+^-ATPase complex. We demonstrated that both Na_X_ and Na^+^/K^+^-ATPase α1 isoform were highly expressed in Neuro2a cells, as shown by immunofluorescence (Figure 3A). An additional control of Na_X_ channel expression has been performed in Neuro2a cells in the presence of different concentrations of the control peptide (Supplemental Figure 1A). As an internal control, we stained for NeuN and MAP2, two recognized post-mitotic neuronal markers (data not shown). To characterize Neuro2a cells as neuronal cells with Na^+^-sensing ability, we also tested their response to voltage step depolarization (Figure 3B), and to hyper- or hyponatremic aCSF application (170 and 100 mM NaCl respectively). Transient application of hyper- or hyponatremic aCSF evoked respectively inward and outward Na^+^ currents (Figure 3C) similar to those observed in dissociated MnPO neurons (Berret et al., 2013). Similar experiments were executed in the presence of strophanthidin, a Na^+^-K^+^-ATPase α-1 isoform inhibitor (40 μM/1 min). We observed that Na^+^-sensing in differentiated Neuro2a cells is partly regulated in by the α-1 isoform of the Na^+^-K^+^-ATPase, as indicated by the strophanthidin-mediated inhibition of the evoked Na^+^ current (Figure 3D). Indeed, strophanthidin application induced a significant reduction of the evoked Na^+^ current (from 17.49 ± 3.35 to 12.69 ± 3.10 pA; Paired *t*-test, *t* = 5.852; *P* = < 0.0001, *n* = 18, Figure 3E).

### Na_X_ and Na^+^/K^+^-ATPase α1 are directly but differently involved in Na^+^-sensing

**Figure 4:**
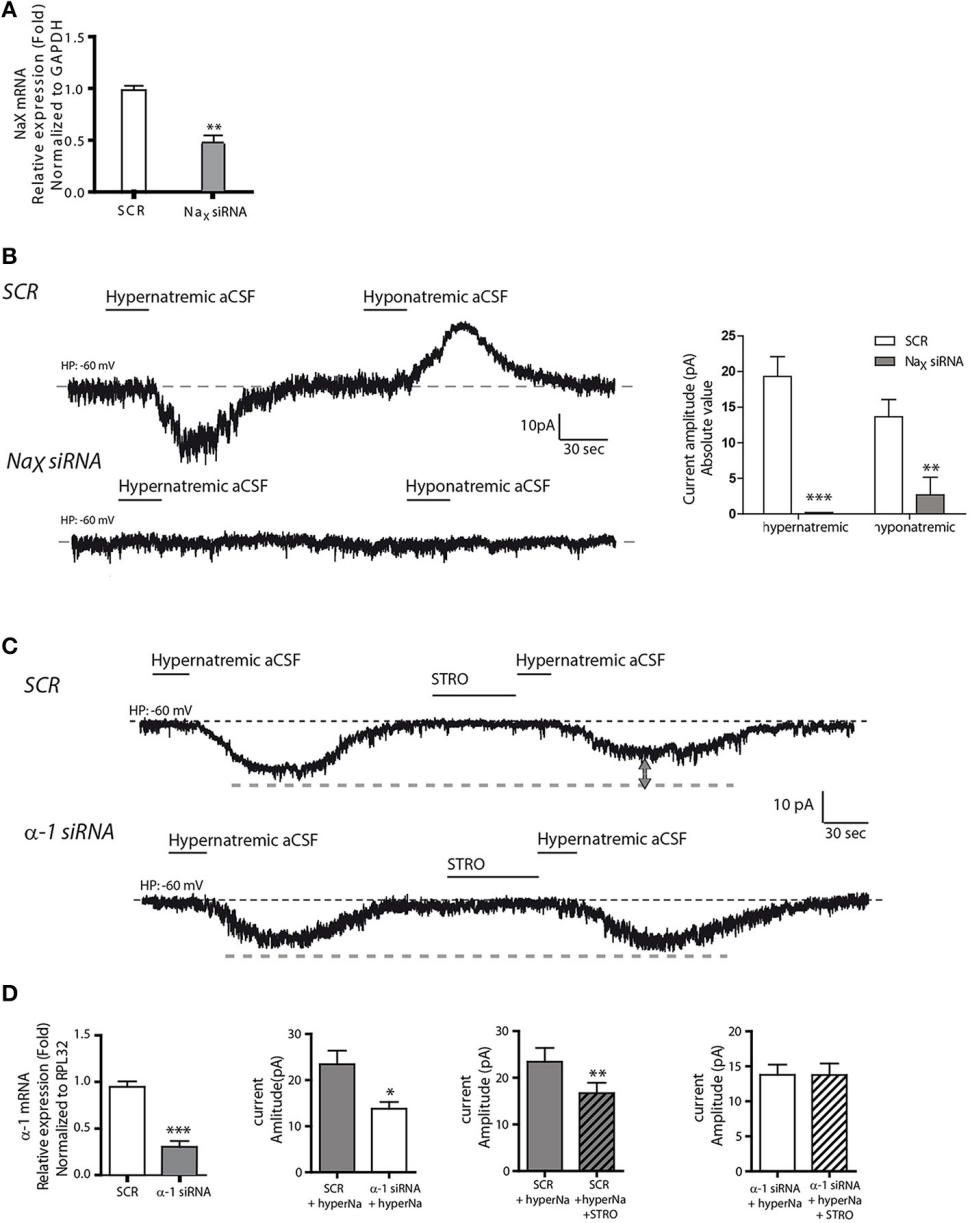
**Na_X_ channel and Na^+^/K^+^-ATPase α-1 isoform are both necessary for integral response and regulation to extracellular Na^+^ change**. **(A)** Relative expression of Na_X_ mRNA after scrambled or Na_X_ siRNA transfection (^**^P < 0.1). We commonly use RPL32 or Gapdh to normalize as these are recognized house-keeping genes. **(B)** Inward Na^+^ current evoked by hypernatremic (170 mM NaCl) and hyponatremic (100 mM NaCl) aCSF in differentiated Neuro2a cell transfected with a scrambled siRNA (top trace). Transfection with Na_X_ siRNA abolished the response to both hypo- and hypernatremic aCSF (bottom trace). Absolute value of Na^+^ current amplitude triggered by application of hyper- or hyponatremic aCSF after SCR or Na_X_ siRNA transfection (^**^P < 0.1; ^***^P < 0.01). **(C)** Strophanthidin-mediated inhibition of inward Na^+^ current evoked by hypernatremic aCSF in differentiated Neuro2a cells transfected with a scrambled siRNA (top trace). Transfection with α-1 isoform siRNA abolished the strophanthidin inhibitory effect (bottom trace). **(D)** Relative level of Na^+^/K^+^-ATPase α-1 isoform mRNA after scrambled or α-1 isoform siRNA transfection (left panel). Basal response triggered by the application of hypernatremic aCSF after scrambled or α-1 isoform siRNA transfection (middle left panel). Na^+^ current amplitude triggered by hypernatremic aCSF under control condition or after strophanthidin application in Neuro2a cell transfected with scrambled (middle right panel) or with α-1 isoform siRNA (right panel) (^*^P < 0.5; ^**^P < 0.1; ^***^P < 0.01).

In a next series of experiments, we performed genetic knockdown experiments to further characterize this Na^+^-sensing system. First, we used short interfering RNAs (siRNAs) against Na_X_ mRNA to investigate the functional consequences of reduced Na_X_ levels. We observed a significant reduction in Na_X_ mRNA levels following siRNA-treatment compared to a scrambled siRNA control sequence (SCR) (1.002 ± 0.026 and 0.538 ± 0.087 for Na_X_ siRNA and SCR respectively; unpaired *t*-test, *t* = 5.061; *P* = 0.0072, *n* = 3, Figure 4A). In addition, our immunostaining experiment showed a significant reduction of Na_X_ immunoreactivity after NaX siRNA treatment compared to SCR (from 0.127 ± 0.043; *n* = 14 for SCR, to 0.071 ± 0.031 *n* = 30 for NaX siRNA; unpaired *t*-test, *t* = 4.365; *P* = 0.0003) (Supplemental Figures 1B,C). In these conditions, electrophysiological recordings showed that Na_X_ downregulation completely abolished the Na^+^ current evoked by hypernatremic aCSF (from 19.33 ± 2.76; *n* = 7 for SCR, to 0.00 ± 0 *n* = 9 for Na_X_ siRNA; unpaired *t*-test, *t* = 8.907; *P* = < 0.0001) (Figure 4B). These findings are in agreement with previous studies linking Na^+^-sensing to the Na_X_ channel (Grob et al., 2004; Tremblay et al., 2011).

Secondly, we used siRNAs against the Na^+^/K^+^-ATPase α1 isoform. By real-time quantitative RT-PCR, we observed a significant reduction in α1 mRNA relative level following siRNA treatment (1.004 ± 0.039 and 0.305 ± 0.044 for α1 siRNA and SCR respectively; unpaired *t*-test, *t* = 11.80; *P* = < 0.0001, *n* = 6 for each condition, Figure 4D). Here, Na^+^/K^+^-ATPase α1 downregulation caused a significant reduction in the inward Na^+^ current triggered by hypernatremic aCSF application. The Na^+^ current amplitude was significantly reduced relative to the scrambled control (from 23.45 ± 2.93 to 13.75 ± 1.45 pA; unpaired *t*-test, *t* = 2.953; *P* = 0.0145, *n* = 6 for each condition, Figures 4C,D). Importantly, we also observed an abolition of the strophanthidin-mediated inhibition of the evoked Na^+^ current. In control conditions, strophanthidin induced a significant reduction of the inward Na^+^ current amplitude from 23.45 ± 2.93 to 16.71 ± 2.20 pA (paired *t*-test, *t* = 5.891; *P* = 0.002, *n* = 6), whereas in α-1 siRNA conditions, there was no change in the Na^+^ current amplitude (13.79 ± 1.4 vs. 13.76 ± 1.65 pA; paired *t*-test, *t* = 0.0479; *P* = 0.963, *n* = 6 for each condition, Figure 4D). Thus, the α-1 isoform of the Na^+^-K^+^-ATPase is necessary to obtain an integral response induced by extracellular Na^+^ change, and to regulate the inward Na^+^ current through the strophantidin.

Taken together, these results demonstrate that differentiated Neuro2a cells provide a good alternative model to study Na^+^ homeostasis regulation under physiological conditions. Therefore, we used this cell line to further study the effects of systemic changes in [Na^+^] on the Na_X_/Na^+^-K^+^-ATPase α-1 isoform complex activity.

### Extracellular [Na^+^] influences Na_X_/Na^+^-K^+^-ATPase α-1 complex activity in neuro2a cells

**Figure 5:**
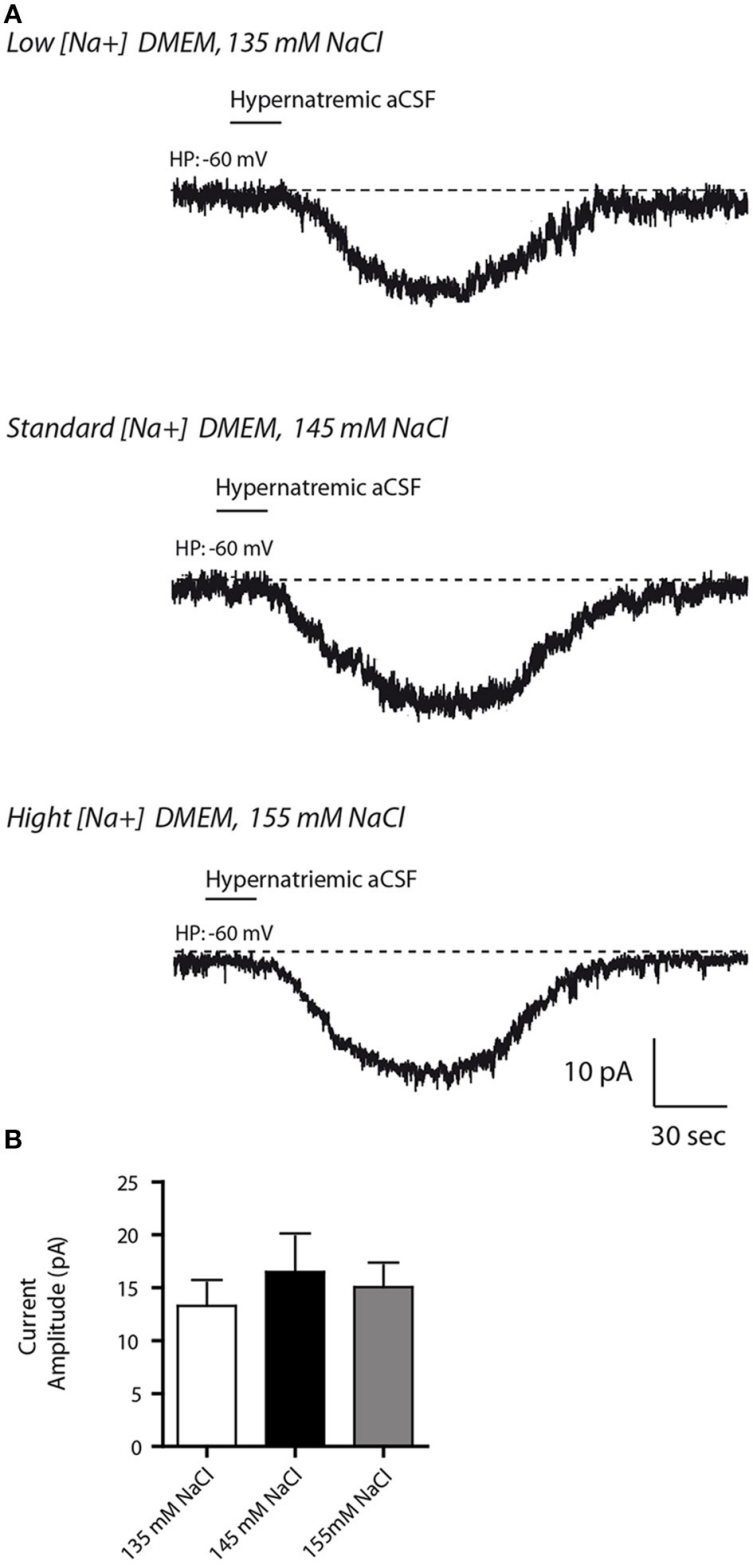
**Altered Na^+^ environment in the culture medium does not have any influence on the basal response to transient change in [Na^+^]_out_ in differentiated Neuro2a cells. (A)** Inward Na^+^ currents evoked by hypernatremic (170 mM NaCl) aCSF in differentiated Neuro2a cell after 48 h incubation in culture medium containing 135 mM (top trace), 145 mM (middle trace,) or 155 mM NaCl (bottom trace). **(B)** Na^+^ current amplitude triggered by application hypernatremic aCSF under these three conditions.

In order to mimic [Na^+^] changes in the CSF induce by diets, we next modulated [Na^+^] levels in the extracellular medium. Specifically, differentiated Neuro2a cells were incubated for 48 h in either 135 mM NaCl to mimic hyponatremic or in 155 mM NaCl to mimic hypernatremic conditions, while control cells were kept in a medium containing 145 mM NaCl. In all conditions, no obvious cell death was observed, as determined by Trypan blue test. Note that extracellular Na^+^ concentrations were determined to obtain a variation range closest to physiological (non-pathological) condition (for review see Verbalis, 2003). We then tested whether hypo- or hypernatremic conditions could modulate the inward Na^+^ current triggered by a transient application of hypernatremic (170 mM NaCl) solution (Figure 5). In all conditions, we observed no significant changes in Na^+^ current amplitude [13.29 ± 6.15, 16.49 ± 3.351, 15.05 ± 2.098 pA for 135, 145, 155 mM respectively; ANOVA *F*_(2, 34)_ = 0.2557; *P* = 0.776, *n* = 12 for each condition]. Thus, the basal mechanism underlying Na^+^-sensing seems not to be affected by change in [Na^+^] in culture medium in a 48 h period.

**Figure 6:**
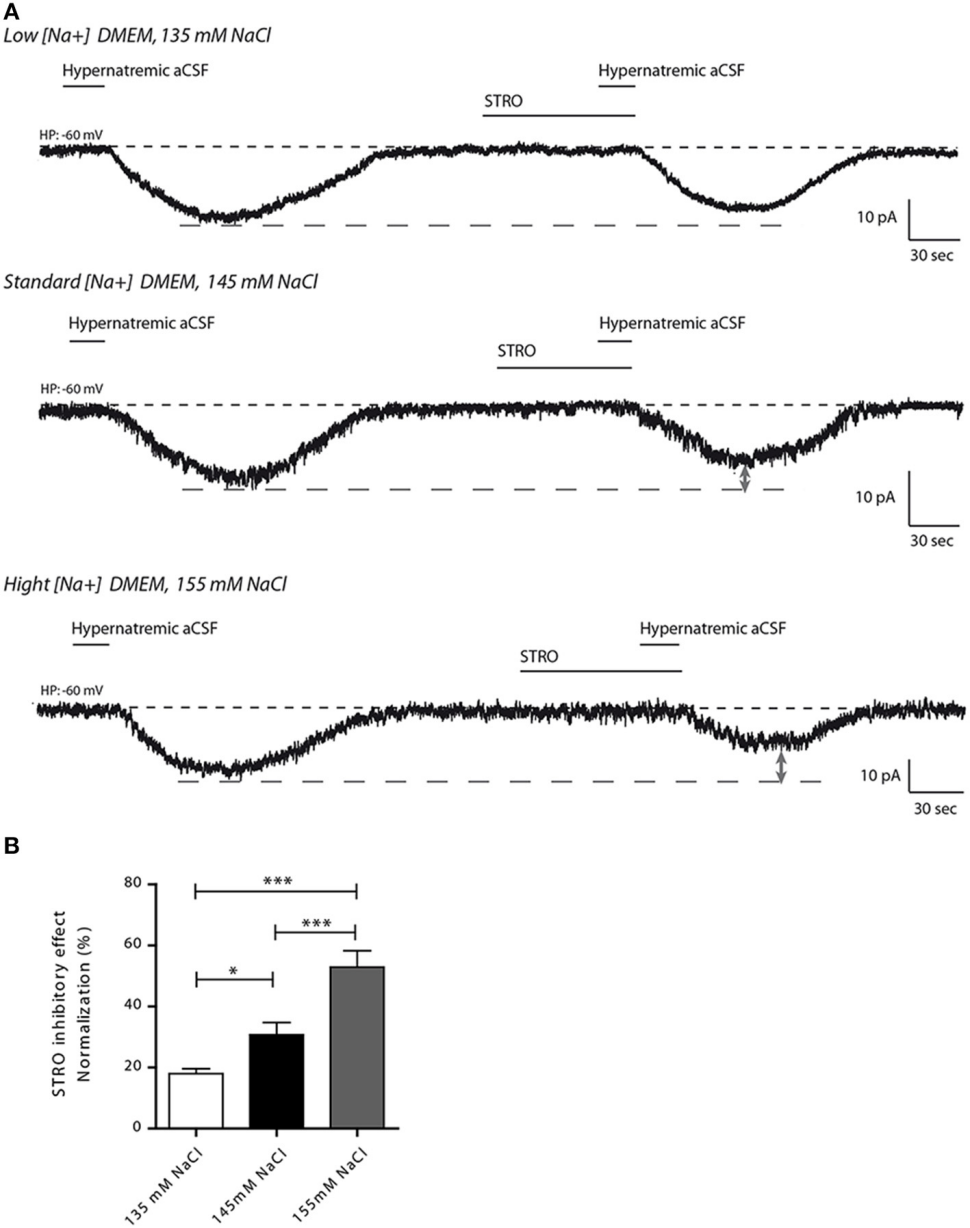
**Altered extracellular Na^+^ concentration influences the inhibitory effect of strophanthidin inhibitory in differentiated Neuro2a cells**. **(A)** Strophanthidin-mediated inhibition of inward Na^+^ current evoked by hypernatremic aCSF in differentiated Neuro2a cells treated with culture medium containing 135 mM (top trace), 145 mM (middle trace) or 155 mM NaCl (bottom trace) for 48 h. **(B)** Bar graph reporting the normalized strophanthidin inhibitory effect on the inward Na^+^ current under the three conditions. (^*^P < 0.5; ^***^P < 0.01).

We next investigated the effects of strophanthidin inhibition on inward Na^+^ current triggered by transient application of hypernatremic aCSF under hyponatremic (135 mM), control (145 mM) or hypernatremic (155 mM) conditions (Figure 6A). A reduction of strophanthidin-mediated inhibition was induced by hyponatremic condition, while an increase of this inhibition was induced by hypernatremic condition [17.97 ± 1.624, 30.71 ± 4.029, 52.86 ± 5.348% for 135, 145, 155 mM respectively; ANOVA *F*_(2,34)_ = 13.32; *P* = < 0.0001, *n* = 12 for each condition, Figure 6B].

Taken together, these results support the results obtained with the dissociated MnPO neurons as the variations in the Na^+^ environment regulate the activity of the Na_X_/Na^+^/K^+^-ATPase complex without affecting the basal Na^+^ sensing.

### [Na^+^] influences Na^+^/K^+^-ATPase α-1 complex colocalization and expression in neuro2a cells

**Figure 7:**
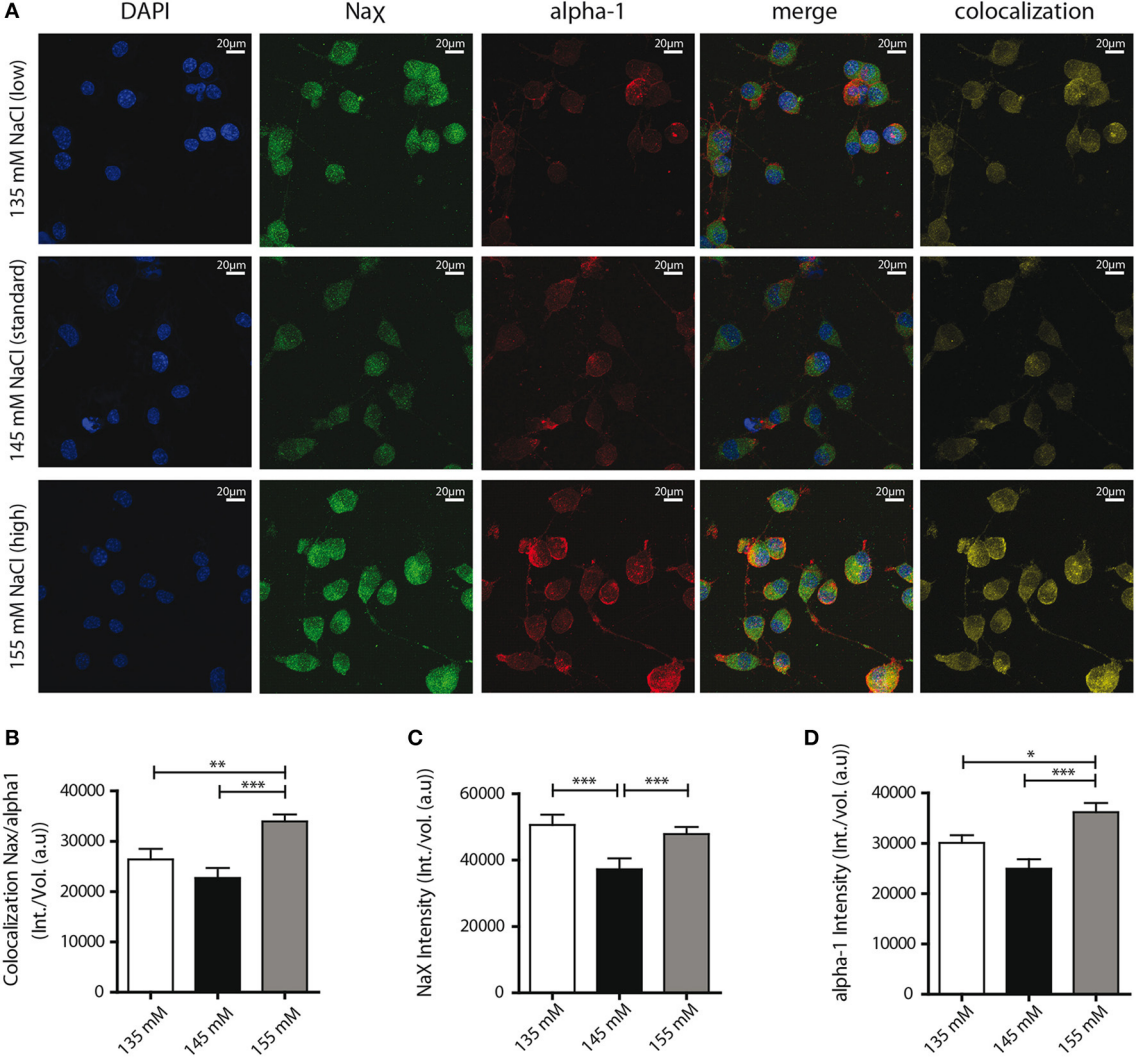
**Confocal detection report variations of Na_X_ and Na^+^/K^+^-ATPase α-1 isoform protein expression and colocalization under the different Na^+^ environments**. **(A)** Representative microphotographs of immunocytochemical staining of differentiated Neuro2a cells. DAPI, Na_X_ channel, Na^+^/K^+^-ATPase α-1 isoform staining and colocalization ratio was analyzed by Bitplane Imaris 7.5.1 software, after treatment with culture medium containing 135 mM (upper line), 145 mM (middle line) or 155 mM NaCl (lower line) during 48 h. Colocalization rate between the two partners **(B)** and relative intensity of Na_X_ channel protein **(C)**, Na^+^/K^+^-ATPase α-1 isoform protein **(D)** under the three conditions. (^*^P < 0.5; ^**^P < 0.1; ^***^P < 0.01).

Finally, we aimed to determine the mechanism underlying the functional changes in Na_X_/Na^+^/K^+^-ATPase activity observed. Our previous studies in dissociated MnPO neurons shown that about 50% of Na_X_ and Na^+^/K^+^-ATPase α-1 isoform protein at the cell membrane were colocalized and formed Na^+^-microdomains (Berret et al., 2013). We suspect that a raise in the colocalization rate of these two partners at the cell membrane could explain the functional modulation of the Na_X_-Na^+^/K^+^-ATPase complex activity induced by environmental changes in [Na^+^]. To address this hypothesis, we used immunostaining to visualize the Na_X_ channel and the Na^+^/K^+^-ATPase α-1 isoform on differentiated Neuro2a cells (Figure 7A). For these experiments, we analyzed colocalization between two proteins within the cell as well as on the cell membrane using the Costes' estimation (Costes et al., 2004) (see Materials and Methods). A significant increase in Na_X_/α-1 isoform colocalization was observed in hypernatremic conditions [24944 ± 1417, 22696 ± 1614, 30804 ± 1323 a.u for 135, 145, 155 mM respectively; ANOVA *F*_(2, 56)_ = 8.964; *P* = 0.0003, *n* = 20, Figure 7B]. To better understand this process, we also examined separately the intensities of Na_X_ and α-1 isoform stainings. We observed a significant increase in Na_X_ signal intensity under hyponatremic and hypernatremic conditions [50587 ± 2698, 37238 ± 2647, 47821 ± 1517 a.u for 135, 145, 155 mM respectively; ANOVA *F*_(2, 56)_ = 9.596; *P* = 0.0002, *n* = 20, Figure 7C], whereas we observed a significant upregulation of α-1 signal intensity only under hypernatremic conditions [30088 ± 1536, 24931 ± 1915, 36198 ± 1821 a.u; ANOVA *F*_(2, 56)_ = 10,41; *P* = 0.0001, *n* = 20, Figure 7D). These results suggest that hypernatremic change in the extracellular environment induces an increase in expression in both Na_X_ channel and Na^+^/K^+^-ATPase α-1 isoform and thus explain that the number of complex is higher at cell surface. Interestingly, during a hyponatremic challenge, increase only in Na_X_ channel expression level can be observed, making an increase in the rate of colocalisation impossible. We assume that this mechanism acts on the ratio between the Na_X_ channel and the Na_X_/Na^+^/K^+^-ATPase α-1 complex. We hypothesis that the boost of the expression of Na_X_ channel without the control of the Na^+^/K^+^-ATPase α-1 isoform, could explain the attenuation of the strophantidin-inhibition observed in the hyponatremic condition.

## Discussion

The present study proposes a novel mechanism of how changes in extracellular [Na^+^] may contribute to the regulation of sodium homeostasis. In the first series of experiments, we were able to demonstrate that environmental changes in Na^+^ modulate the activity of the Na_X_/Na^+^/K^+^-ATPase α-1 isoform complex in isolated MnPO neurons. In the second, complementary series of experiments, we established a new cellular model in order to better understand the molecular mechanism(s) involved in Na_X_/Na^+^/K^+^-ATPase α-1 isoform complex activity regulation.

### Alteration in Na^+^ sensing observed *in vivo* could be modeled in neuro2a cells

Electrophysiological results obtained about the functional activity of the Na_X_/Na^+^/K^+^-ATPase α-1 isoform complex are very similar in Neuro2a cells and in MnPO neurons. Data obtained from both immortalized cells and isolated cells from rats demonstrated that evoked Na^+^ current amplitude does not change under different Na^+^ conditions or after salt-diet, indicating that the mechanism of Na^+^-sensing is not affected. In addition, we showed that current induced by hypernatremic and hyponatremic aCSF was completely abolished by Na_X_ siRNA treatment, suggesting that the Na_X_ channel is responsible in most part for this response. While only a partial (i.e., ~50%) reduction of total Na_X_ mRNA levels was observed in these conditions, we cannot exclude that Na_X_ protein exists in various functional pools in the cell, some of which might be more prone to siRNA treatment (i.e., faster or slower turnover). This hypothesis is in line with our cell surface experiments and rationally correlates with our electrophysiological recordings. Together, these findings are in line with previous studies linking Na^+^-sensing to the Na_X_ channel (Grob et al., 2004; Tremblay et al., 2011).

Similarly, we were able to observe an inhibition during the strophanthidin-application in both Neuro2a cells and MnPO neurons. While the basal strophanthidin inhibitory effect is lower in Neuro2a cells than in MnPO neurons (~30 and ~50% respectively), the range of variations observed under the different condition of systemic [Na^+^] are similar. ~13 and ~20% reduction for Neuro2a cells and MnPO neurons was respectively observed under hyponatremic conditions, while ~22 and ~15% augmentation under hypernatremic conditions. Thus, Neuro2a cells show the same Na^+^-sensing properties as MnPO neurons and can reliably be used to study the molecular changes caused by modifications in extracellular [Na^+^] in MnPO neurons.

### Mechanism underlying the modulation of the Na_X_/Na^+^/K^+^-ATPase complex activity during hypernatremic change in the cellular environment

Our findings suggest colocalization of Na_X_ and Na^+^/K^+^-ATPase α-1 increases under extracellular hypernatremic conditions. We propose that these partners form an active complex involved in the activity changes observed in electrophysiology. It's noteworthy that the Costes' estimation used in this study is specifically used to generate an artifact-free colocalization channel between 2 signals (proteins) of interest (see Materials and Methods).

Electrophysiological recordings under these conditions show an up-regulation of the strophanthidin-mediated inhibition corresponding to an increase in the α-1 isoform regulatory activity on the Na_X_ channel, which is consistent with an increase in protein expression. Thus, we suggest that both an increase in colocalization and expression could represent the molecular phenomenon underlying functional changes observed in electrophysiology under hypernatremic conditions, namely the increase of strophanthidin inhibition observed in the hypernatremic condition.

#### Mechanism underlying the modulation of Na_X_/Na^+^/K^+^-ATPase complex activity, during hyponatremic change in the cellular environment

Under extracellular hyponatremic conditions in Neuro2a cells, we observed no significant changes in either α-1 isoform expression, or in the level of Na^+^/alpha-1 colocalization. On the other hand, Na_X_ channel protein expression was increased. Since environmental [Na^+^] is lower in this experimental context, we assume that a raise of Na_X_ channel expression could be a reliable mechanism to ensure the correct detection of [Na^+^].

Paradoxically, our electrophysiological recordings show a reduction in Na_X_/Na^+^/K^+^-ATPase complex activity in both experimental models we investigated. We hypothesize as Na_X_ channel expression is increased, there is more “free” Na_X_ channels at the cell membrane relative to the α-1 isoform. Thus, there is less α-1 isoform available to regulate Na_X_ channel activity. Therefore, an increase in the number of Na_X_ channels at the membrane could ensure proper extracellular Na^+^-detection, whereas the regulatory activity of the Na_X_/Na^+^/K^+^-ATPase is proportionally reduced. This combined regulatory mechanism likely functions as a sensitive mediator of Na^+^ variation.

### Physiological relevance and general conclusions

Taken together, our study demonstrates that changes in systemic [Na^+^], induced by low- or high-salt intake, modulate cellular Na^+^ homeostasis regulation by altering the activity of the Na_X_/Na^+^/K^+^-ATPase α-1 isoform complex. In Neuro2a cells, this regulation occurs via the modulation of the number and the ratio of Na_X_/Na^+^/K^+^-ATPase α-1 complexes expressed on the cell membrane surface. Hypernatremic change in extracellular environment induces an increase in the functional activity of the complex, whereas hyponatremic change reduces its functional activity.

In our rat model, the complex activity modulation during environmental challenges could influence the excitability of MnPO neurons. We suppose that altered neuronal excitability can, in turn, regulate information transfer from MnPO neurons to their targets, mainly to magnocellular neurons of PVN and SON in charge of vasopressin and oxytocin release (Antunes-Rodrigues et al., 2004). These hormones are involved in renal Na^+^ and water reabsorption, as well as in behavior related to the control of salt and water intake (Tanaka, 1989; McKinley et al., 1999a,b).

Of course, we cannot exclude the implication of additional factors triggered by sodium diet that could be involved in this process. For example, high sodium challenge can trigger various physiological responses such as hormone secretion. Aldosterone, one of these hormones, is able to bind to the mineralocorticoid receptors (MR) and regulate cytoplasmic or nuclear mechanisms in order to regulate targeted protein expression (Pietranera et al., 2001; Thomas and Harvey, 2011; Dooley et al., 2011). Further studies are required to elucidate the specific role of those potent factors during Na^+^ challenge.

### Conflict of interest statement

The authors declare that the research was conducted in the absence of any commercial or financial relationships that could be construed as a potential conflict of interest.

## Supplementary material

Supplemental Figure 1 **Na_X_ antibody specificity and expression, and Na_X_ siRNA efficiency in Neuro2a cells**. **(A)** DAPI staining and immunocytochemical visualization of Na_X_ channel and in differentiated Neuro2a cells after treatment with 0, 0.1, 1.0, 5.0, and 10.0 μg of control peptide. **(B)** Representative confocal microphotographs of immunocytochemical staining of differentiated Neuro2a cells. DAPI and Na_X_ channel staining intensity was analyzed using NIH ImageJ software (v1.49), after after transfection with scrambled siRNA or Na_X_ siRNA. **(C)** Bar graphs reporting the relative intensity of Na_X_ channel immunoreactivity in Neuro2a cells transfected with scrambled siRNA or Na_X_ siRNA. (^***^*P* < 0.01).
