# Supplementary figures and images for: Extracellular Na+ levels regulate formation and activity of the NaX/alpha1-Na+/K+-ATPase complex in neuronal cells
Source: Front Cell Neurosci. 2014 Dec 4;8:413. doi: 10.3389/fncel.2014.00413 (PMC4255601; doi:10.3389/fncel.2014.00413)

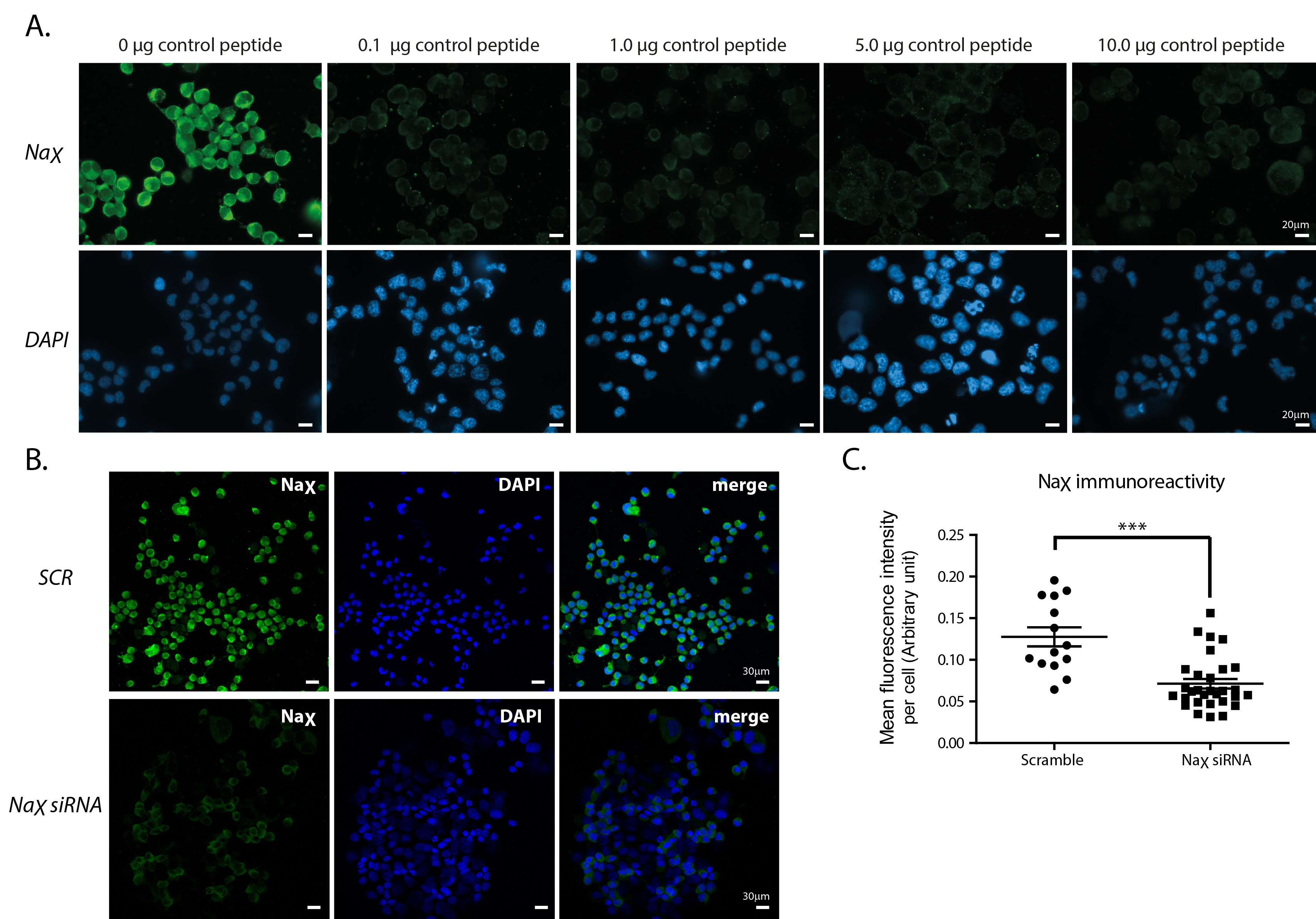

Supplement: Supplemental Figure 1 — NaX antibody specificity and expression, and NaX siRNA efficiency in Neuro2a cells. (A) DAPI staining and immunocytochemical visualization of NaX channel and in differentiated Neuro2a cells after treatment with 0, 0.1, 1.0, 5.0, and 10.0 μg of control peptide. (B) Representative confocal microphotographs of immunocytochemical staining of differentiated Neuro2a cells. DAPI and NaX channel staining intensity was analyzed using NIH ImageJ software (v1.49), after after transfection with scrambled siRNA or NaX siRNA. (C) Bar graphs reporting the relative intensity of NaX channel immunoreactivity in Neuro2a cells transfected with scrambled siRNA or NaX siRNA. (***P < 0.01). [file Image1.JPEG]
